# Turing pattern design principles and their robustness

**DOI:** 10.1098/rsta.2020.0272

**Published:** 2021-12-27

**Authors:** Sean T. Vittadello, Thomas Leyshon, David Schnoerr, Michael P. H. Stumpf

**Affiliations:** ^1^ School of BioSciences, University of Melbourne, Melbourne, Victoria 3010, Australia; ^2^ School of Mathematics and Statistics, University of Melbourne, Melbourne, Victoria 3010, Australia; ^3^ Department of Life Sciences, Imperial College London, London, UK

**Keywords:** pattern formation, positional information, design principles

## Abstract

Turing patterns have morphed from mathematical curiosities into highly desirable targets for synthetic biology. For a long time, their biological significance was sometimes disputed but there is now ample evidence for their involvement in processes ranging from skin pigmentation to digit and limb formation. While their role in developmental biology is now firmly established, their synthetic design has so far proved challenging. Here, we review recent large-scale mathematical analyses that have attempted to narrow down potential design principles. We consider different aspects of robustness of these models and outline why this perspective will be helpful in the search for synthetic Turing-patterning systems. We conclude by considering robustness in the context of developmental modelling more generally.

This article is part of the theme issue ‘Recent progress and open frontiers in Turing’s theory of morphogenesis’.

## 1. Introduction

In 1952, Alan Turing published the second of his groundbreaking papers, *The Chemical Basis ofMorphogenesis* [1]. His ambition was to present a mechanism capable of producing patterns using purely biochemical processes, without any further need for mechanical or external influences. In short, he was trying to understand how biological patterns could arise through self-organization. It was an ingenious attempt to solve an obvious but poorly understood problem in developmental biology. A mere six references—Michaelis and Menten being the only paper published in a journal, with the other five being books—mainly served to lay out the problem, but contained little in terms of prior work on which Turing could build.

We can contrast this with another popular mechanism proposed to explain spatial patterns in development, namely positional information, also known as the *French Flag Model*. Its most vocal proponent was Lewis Wolpert whose 1969 paper [2] contained ample references, in contrast to Turing’s paper, including references to extensive earlier experimental work; some of this experimental work presaged the same positional information mechanism on which Wolpert elaborated [3].

Compared to the Turing pattern (TP) model, positional information (PI) was immediately and enthusiastically received by the biological community [4]. By contrast, Turing’s work, and reaction–diffusion systems more generally, have been hugely influential for the development of mathematical biology. Among biological audiences, however, even including many pioneers of theoretical biology, TPs were not accepted and generally dismissed as unhelpful. One of the appeals of PI may have been that it is a very easy mechanism to describe and understand, unlike the self-organizing mechanism of TPs, which arguably really require the analysis of mathematical models. Not surprisingly, perhaps, early papers proposing PI mechanisms were very light on mathematical or mechanistic detail, and contained very little concrete information on the origins of morphogen gradients.

The past decade has seen renewed interest in TPs, including ample experimental evidence for their existence in real biological systems (a brief overview is given below). There has also been increased interest in understanding more completely the types of biological systems that could give rise to TPs [5]. This search for so-called *design principles* is inspired by attempts to engineer TPs *in vivo* using tools from synthetic biology [6]. Such a *de novo* and rationally engineered system would arguably be a major step towards understanding the role of TP mechanisms in developmental biology. Moreover, it would open up exciting opportunities in tissue engineering, regenerative medicine and biotechnological applications, where self-organized reproducible patterns are required.

Here we discuss an aspect of model development in biology that is essential for confronting the beauty of mathematical models with the ugly truth of reality: the extent to which the assumptions underlying our models are robust and in line with what we see in nature [7]. Intuitively, if a model is not robust then even slight changes in the model parameterization or model structure can give rise to different model behaviour corresponding to variance of biological function. These differences can be of a quantitative or of a qualitative nature. As models will always differ from reality—by design as well as necessity—lack of robustness of a model’s predictions should temper our trust in a model as a description of reality.

We will explore facets of the robustness of the TP mechanism, and for this we first discuss the mathematical hallmarks of TP mechanisms. We then provide a brief overview of how experimental studies of TPs reflect these basic principles. Having established the existence of TPs in nature, we then provide perspectives on robustness. After discussing TPs and TP-generating mechanisms from these perspectives, we revisit the competing mechanisms of TPs and PI and argue that this dichotomy has in the past been overblown. Both mechanisms have their place and roles to fill in developmental biology; often, indeed, they may be required simultaneously [8]. Furthermore, at a deeper mathematical level we can also show that mathematical models of these two mechanisms are in fact closely related.

## 2. Turing-pattern mechanism

**Figure 1.**
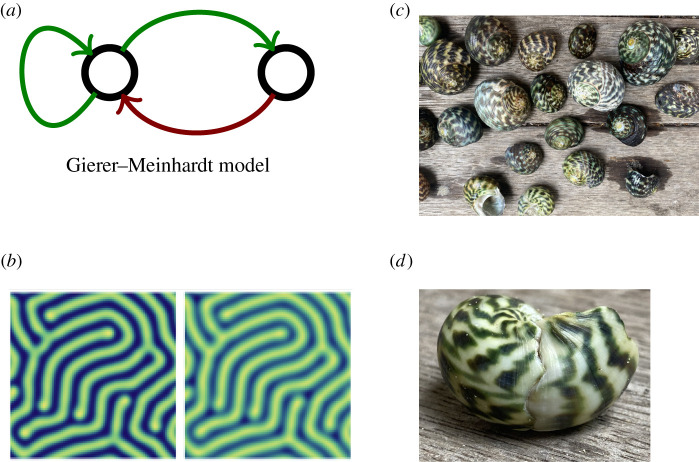
Representations and manifestations of Turing patterns. (*a*) The Gierer–Meinhardt model is one of the most widely studied systems producing Turing patterns, and it embodies the local auto-activation and lateral inhibition mechanisms that are the hallmarks of the majority of Turing-pattern mechanisms. (*b*) An example of a Turing pattern produced by the Gierer–Meinhardt system; here the two morphogen patterns are in phase, which means the areas of high and low concentrations of both morphogens coincide. (*c*) Patterns on snail and sea shells are one of the classical examples of Turing patterns in nature.(*d*) A sea shell illustrating the robustness and self-repairing ability of Turing-pattern systems. At some stage during growth this shell must have cracked and interrupted the patterning. The Turing-pattern mechanism appears to have set in quickly afterwards and a pattern is seen to reappear (to the right of the crack). [Shells were provided by H. X. Stumpf Laidlaw]. (Online version in colour.)

Turing’s original mechanism involves two morphogens, A and B, which act as illustrated in figure 1*a*: local activation (of A by itself, and of B by A via diffusion), followed by lateral inhibition (of A by B, again via diffusion).

In mathematical terms, we have a range of options for modelling such a scenario, and here we follow the model proposed by Gierer & Meinhardt [9] which is embodied by the system of equations,

2.1 $$\frac{\partial A}{\partial t}=D_{A}\nabla^{2}A+\frac{\rho A^{2}}{B(1+\mu_{A}A^{2})}-k_{A}A+\rho_{A}$$

and

2.2 $$\frac{\partial B}{\partial t}=D_{B}\nabla^{2}B+\rho A^{2}-k_{B}B+\rho_{B},$$

with diffusivities $D_{A}$ and $D_{B}$, basal production rates $\rho_{A}$ and $\rho_{B}$, degradation rates $k_{A}$ and $k_{B}$, saturation constant $\mu_{A}$ and source density ρ. As this is a system of partial differential equations we also need to specify the boundary conditions, which are here taken to be zero flux across the domain boundaries.

A network representation of the Gierer–Meinhardt model is shown in figure 1*a*, and an example pattern in the morphogens A and B produced with the model is shown in figure 1*b*. The TP mechanism can give rise to different patterns, including dots, stripes, and more complicated labyrinth structures. It is worth remarking here that knowledge of the existence of a Turing instability does not immediately suffice to predict what type of pattern is produced. The same network—as opposed to the fully specified and parameterized dynamical system—can give rise to different patterns. What has been emerging from recent surveys of TPs is that (auto-) activation and (lateral) inhibition appear to be requirements for the formation of TPs; certainly both morphogen models that have been found to produce TPs contain such a core motif; and it is also near ubiquituous among models of three morphogens.

This simple activation-inhibition architecture gives rise to a TP under certain conditions. Consider a general reaction–diffusion system for morphogens A and B as representing a candidate TP model,

2.3 $$\frac{\partial A}{\partial t}=D_{A}\nabla^{2}A+f(A,B)$$

and

2.4 $$\frac{\partial B}{\partial t}=D_{B}\nabla^{2}B+g(A,B),$$

where f(A,B) and g(A,B) are the reaction terms. The first required condition is that the fixed point of the non-spatial system where the dynamics with diffusion are set to zero,

2.5 $$\frac{\text{d}A}{\text{d}t}=f(A,B)$$

and

2.6 $$\frac{\text{d}B}{\text{d}t}=g(A,B),$$

must be stable in the sense that the Jacobian matrix corresponding to equations (2.5) and (2.6),

2.7 $$J=\left(\begin{array}{cc} \frac{\partial f}{\partial A} & \frac{\partial f}{\partial B}\\ \frac{\partial g}{\partial A} & \frac{\partial g}{\partial B} \end{array}\right),$$

must have all eigenvalues with negative real part at the fixed point [10]. If the real part of the leading eigenvalue becomes positive when diffusion is turned on ($D_{A},D_{B}>0$), that is, if the fixed points become unstable in the diffusive system, then we speak of a Turing instability—more precisely a Turing type 1 instability, as there are other types of instabilities which are discussed in the literature [11]—and we expect to see the formation of a spatially extended TP.

It is therefore important to keep in mind that whenever we speak of a TP system we refer to a mathematical model that exhibits a TP for only restricted ranges of the model parameters. In the notation of the Gierer–Meinhardt model (2.1) and (2.2), a TP is observed for a set of reaction parameters $\theta_{0}=(\rho_{A},\rho_{B},k_{A},k_{B},\mu_{A},\rho )$ and a set of diffusion parameters $\theta_{D}=(D_{A},D_{B})$ when $\theta =(\theta_{0},\theta_{D})\in \Omega_{\theta_{0}}$, which is the subset of the whole permissible parameter space $\Omega_{\theta }$ for which TPs are observed for the model. Finding parameter regions $\Omega_{\theta_{0}}$ for which TPs are observed can be computationally demanding [11].

In natural systems, TPs have been implicated in a range of contexts where periodic or regular spatial arrangements are required. Published examples include: the formation of limbs, digits and vertebrae [12]; skin pigmentation patterns in zebrafish [13]; hair follicle and feather development in mice and chicks[14], respectively; and, perhaps more trivially, the pattern on seashells and snails (beautifully discussed in [15]; see also figure 1*c*,*d*). The biological hallmark of such systems is that the patterns arise in a self-organized fashion without any obvious or apparent external control, except that over developmental time-scales the expression of genes and the downstream translational activity patterns are orchestrated by intricate signalling processes. In addition to being self-organizing, TPs are also self-repairing, for example see figure 1*d*. This, among other appealing facets, makes them a popular target for synthetic biology, albeit with thus far limited success.

The function of TPs, their potential uses in synthetic biology and regenerative medicine, and their evolutionary role in perhaps driving the origin of multi-cellularity, are closely linked to questions of robustness. These questions are the main topic of the next three sections. We first present a pragmatic and phenomenological approach, before discussing a more rigorous mathematical perspective. This in turn is then used to discuss the relationship of Turing’s mechanism to another developmental-biology mechanism, the PI (*aka* ‘French Flag’) model.

## 3. Robustness: a pragmatic perspective

Robustness means, in very general terms, that a model’s predictions remain largely unchanged when aspects of the model—parameters, details of the model structure, external influences—are changed. Typically, changes to the model are local and not severe, but this does not have to bethe case.

In the context of TPs, we can usefully distinguish between parametric robustness—we consider parameters related to reactions, and parameters related to diffusion, separately—and structural robustness. We also consider two additional types of robustness: robustness of a model to different mathematical modelling or simulation approaches; and robustness of a mechanism to being embedded in a larger network without having its performance compromised.

### (a) Parametric robustness

To estimate parametric robustness we can simply explore the size of the parameter space of the non-diffusion (intracellular) parameters for which we can obtain a single stable stationary state (in [11], we found that bi-stable and multi-stable systems require further analysis), which can become unstable for suitable choices of the diffusion parameters; the fraction of the parameter space for which this is the case is denoted by $\rho_{0}$. The fraction of the diffusion parameter space that renders a stable state unstable once diffusion has been turned on, $\rho_{D}$ is then a measure of the intercellular robustness. Exploring the parameter spaces for $\theta_{0}$ and $\theta_{D}$ separately can reduce the computational burden compared to a joint analysis. It is important to remember that these robustness values will depend on the total parameter space $\Omega_{\Theta }$ that is analysed, and hence may vary between different analyses.

An alternative ansatz to parametric robustness that is especially useful if we are dealing with inferred model parameters—in the case of TPs we are probably still far away from being able to do this—is to take a given parameter value, $\theta^{\prime }$, for which we observe a TP, and perturb it by adding a small random vector δ. We then determine if the model also exhibits a TP, more precisely a Turing instability, for $\theta^{\prime }+\delta$.

### (b) Structural robustness

For the structural robustness analysis [16] we can, for example, look at the network architecture of a model $M^{\prime }$. We can then consider networks within a given graph-edit distance from $M^{\prime }$ to consider which of these networks are themselves capable of producing TPs. The fraction of network architectures that can be reached by, for example, one change to the network (such as adding or removing an edge, or changing the nature of an existing interaction) is then a measure for the structural robustness of model $M^{\prime }$ and denoted here by $\rho_{T}$.

Structural robustness has received comparatively little attention compared to parametric robustness. The reasons for this are two-fold: the bulk of the analyses have only considered a single model at a time, which nearly always precludes considering the effects of changes to model structures. More mundanely, the second reason is that structural robustness or sensitivity analysis requires the numerical analysis of many different models and, for each model, potentially the exploration of vast parameter spaces. Unless suitable numerical approximations are used the computational cost of such analyses quickly becomes prohibitive.

### (c) Total (parametric+structural) robustness

The overall robustness of a TP mechanism depends on the interplay between parametric and structural robustness. We can estimate the robustness of a given model, $M^{\prime }$, by

3.1 $$\rho (M^{\prime })=\rho_{\theta_{0}}\times \rho_{\theta_{D}}\times \rho_{T}.$$

**Figure 2.**
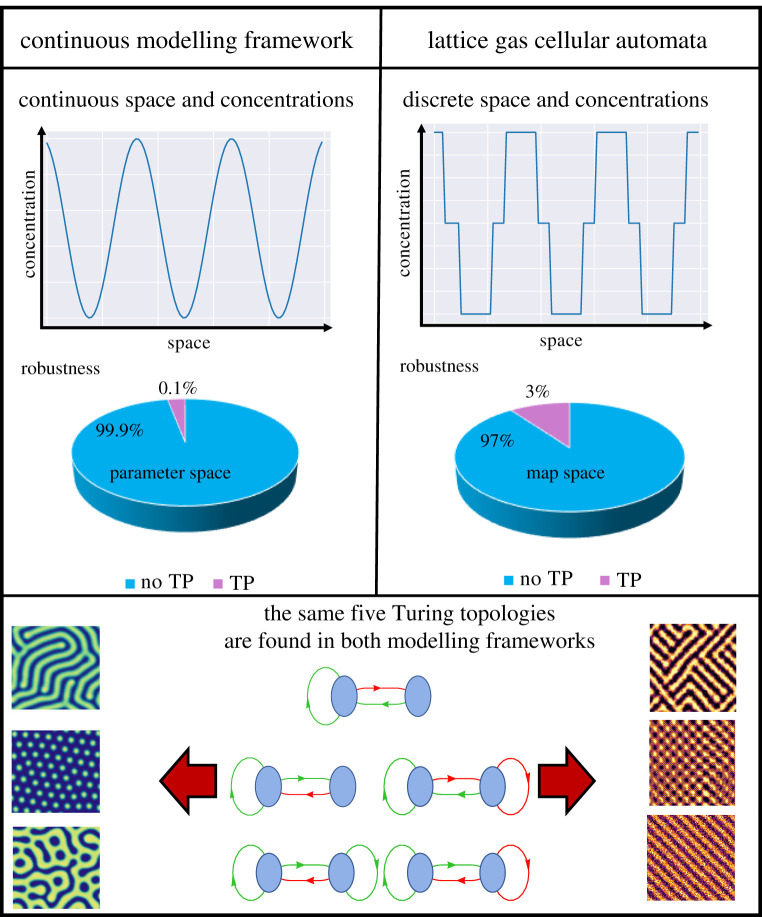
Schematic showing the findings of Scholes *et al.* [11] on the left, and the LGCA model on the right. The continuous modelling framework found a value of 0.1% for intracellular (reaction-parameter space) robustness while the LGCA found a value of 3% for intracellular robustness (reaction-map space). The figure shows the same five Turing topologies found to produce patterns across both models. These were the only five topologies found in both studies to produce patterns. To either side of the Turing topologies are samples of the types of patterns each model is capable of producing in two dimensions. (Online version in colour.)

When we compare the relative roles of parametric and structural robustness we find that for 3-node networks (see figure 2 and [11]) many network architectures are capable of producing TPs, but only for very limited parameter ranges. Such comprehensive analysis is computationally challenging. It does, however, appear to be necessary in order to ensure that the correct lessons about design principles underlying TPs can be distilled.

This suggests that evolutionary processes are likely to generate molecular interaction networks that are in principle capable of giving rise to TPs; but these may in practice be sensitive to parametric variation. This potential ‘easy come, easy go’ aspect of TPs probably deserves some closer attention in the search for synthetically engineered TPs [17].

### (d) Modelling robustness

Yet another, less well understood and less frequently employed form of robustness comes from obtaining similar results from different modelling formalisms [7]. Superficially this seems plausible, but it is important to remember that failure of one modelling formalism to reproduce the results of another formalism—for example stochastic versus deterministic analysis—does not imply that the analysis of one formalism is not reliable: in many situations, some modelling approaches are appropriate while others simply are not.

Turing processes are intrinsically spatial processes, and the spatial or cellular architecture needs to be modelled explicitly. Any non-spatial model would clearly and trivially be inappropriate. But we may, for example, be interested to determine if the design principles found in deterministic analyses are also able to generate TPs if dynamics are stochastic [18]; if the models include discrete time-delays [19], or if the continuous models are replaced by cellular automaton models. The latter has recently been achieved and the results are summarized in figure 2. Reassuringly, here the dynamics of a lattice gas cellular automaton give rise to TPs for essentially the same types of mechanisms as for the continuous case. Such an analysis that compares different modelling frameworks requires careful identification of equivalent model features. A large scale analysis that compared lattice gas cellular automaton models of TPs with continuous models [20] has recently confirmed that only a small number of network architectures are capable of generating stable spatial patterns. This means that design principles are robust with respect to a change in the modelling formalism employed.

This type of robustness is qualitatively distinct from the other two discussed above. The network models are, to all intents and purposes, the same, but their mathematical analysis or simulation employs different methodologies and formalisms. Nevertheless, we can draw some information from this type of robustness about the likely behaviour of real-world systems: natural systems will differ from the assumptions underlying both discrete and continuous models. So if a model behaves similarly under these different scenarios we may have increased confidence in our model offering an acceptable description of the real systems.

### (e) Embedding robustness

Finally, we deal with a subtle problem affecting systems biology models much more generally. In most cases, we model small subsystems of a cell, tissue, organism or ecosystem. In cell biology, we study, for example, signalling or metabolic pathways, or the control of gene regulation mechanisms and their effects of transcript abundance. Rarely, though, do we analyse a system comprehensively. In the context of TPs, for example, we focus on a small number of morphogens and their cellular effects in a very limited domain and context. We ignore a host of other factors, many of which would also have concrete roles to play in tissue formation and biological patterning. Furthermore, if the physiological context affects any of the genes whose products are involved in morphogen production, transport, or degradation, then it is likely to affect the reaction scheme and its kinetics. Finding a TP in isolation, or indeed *in vitro*, is therefore no guarantee that we will see *in vivo* realisations of TPs.

What is required is a measure for a molecular network motif being able to maintain a function (such as the ability of generating a TP) when placed, or embedded, in a physiological context. To our knowledge such a measure does not (yet) exist. Heuristically it seems plausible that a combination of parametric, structural, and modelling robustness may increase our belief that a given design might also work *in vivo*, but we have neither mathematical evidence, nor experimental verification.

## 4. Design principles of robust Turing networks

Due to high computational costs, the analysis of Turing networks and their robustness has been restricted to case studies of single systems and their parametric robustness for a long time [5,21]. Only in recent years have advances in computational and numerical methods allowed more systematic large-scale studies analysing large numbers of reaction networks [11,22–24]. While some of these consider linear reaction terms that simplify the mathematical analysis [23,24], others consider biologically more realistic but harder to study Hill-type reaction terms [11,22]. Zheng *et al.* [22] used activating competitive and inhibiting non-competitive interactions, while Scholes *et al.* studied systems with either all competitive or all non-competitive interactions. While parametric robustness is studied in [22–24], the authors in [11] also analysed structural robustness (cf. §3).

These and other recent studies have greatly enhanced our understanding of general principles underlying Turing networks and allowed for the relaxation of certain constraints that were previously assumed to apply in general. One crucial finding is that the constraint of differential diffusivity can be relaxed. Previous studies found that the inhibitor needs to diffuse substantially faster than the activator [9,25] ($D_{B}\gg D_{A}$, cf. the discussion below equation (2.2)) which raises the question of how biological systems could implement such mechanisms since morphogens typically do not have diffusion rates differing over orders of magnitudes. In [26,27], it has been found that adding a third non-diffusing species to the classical activator-inhibitor model allows for equal diffusivities. This has been verified in [23] for larger numbers of linear systems and in [11] for larger numbers of networks with Hill-type interactions.

In terms of the overall parametric robustness, these large-scale studies have confirmed previous case studies [21,28], that networks produce Turing instabilities only in small fractions of parameter space, even for the most robust networks [11,22]. To design 3-node networks with a larger parametric robustness, the authors in [22] suggest including the classical 2-node activator-inhibitor motifs and to add additional interactions complementing these. In [23], it was found that networks without diffusivity constraint tend to also be more robust with respect to kinetic parameters than networks with constraints. In [11], it has been found that while competitive interactions appear to give rise to a smaller structural robustness than non-competitive ones, they tend to have a larger total robustness. In terms of the number of networks capable of producing Turing instabilities for certain parameter regimes, Zheng *et al.* [22] found substantially more 3-node Turing networks than Marcon *et al.* [23] this likely reflects the importance of the specific choices made for regulatory functions. Scholes *et al.* [11] found even more 3-node Turing networks through finer sampling of the parameter space [11]; this study further identified two core motifs whose combined presence was found to constitute an almost necessary and almost sufficient condition for Turing instabilities to occur.

Most of these studies consider Turing instabilities and appear to assume that every Turing instability leads to a pattern when simulating the system. However, it has recently been found that this is not necessarily the case in multi-stable networks, therefore with multiple stable steady states [11,29]. It has been found that a small number of such systems give rise to spatially homogeneous solutions corresponding to a stable steady state not possessing a Turing instability [11]. Similarly, all these studies do not consider the amplitude of a pattern arising in simulations. We found empirically that many networks give rise to patterns with tiny amplitudes for certain parameter regimes (results not shown). It is questionable how relevant such patterns are for biological systems that are always subject to noise. We therefore argue that more importance should be given to the actual outcomes of simulations in future studies, rather than just the existence of Turing instabilities.

It is also important to note that many biological tissues are subject to domain growth, which is not considered in studies discussed so far. Domain growth was found to substantially change the patterning behaviour of certain systems [30], such as leading to certain networks exhibiting Turing instabilities which do not do so on fixed domains [31], and increasing networks’ parametric robustness [5]. In [32], Konow *et al.* studied the effect of domain growth on the type of patterns emerging, both experimentally and computationally. In [33], the authors studied the role that the speed of growth plays. The conditions for Turing instabilities to occur on growing domains have recently been analysed more generally in [34]. It will be important to see how these results generalize when applied to larger numbers of networks.

Physiological processes typically have associated lag times, or time delays, between the initiation of the physiological mechanism and the resulting functional output [35]. In the context of TPs, gene expression is subject to often considerable delays through the processes of transcription and translation [19]. Mathematical models with time delays can produce a better correspondence with reality, though possibly with an increased difficulty in analysing and simulating the model. Incorporating time delays into TP models can result in major changes in structural robustness, in particular the formation of a pattern may have a highly dependent and complicated relationship with the delay. In [19], the authors consider a reaction–diffusion model with a discrete time-delay associated with the process of gene expression, for both stationary and spatially uniform exponentially-growing domains. It is observed that the delay can considerably increase the time required for the formation of a stable pattern, while on rapidly growing domains the delay may result in the absence of a pattern. Further studies, again using models with discrete time-delays, reinforce the observation that structural robustness has a complicated dependence on the delay [36–39]. It would be of considerable interest to investigate TP models with distributed delays, which are the more realistic continuous analogue of discrete delays, to ascertain their effect on structural robustness.

All studies mentioned in this section so far are based on deterministic reaction–diffusion processes modelled by partial differential equations, and hence by themselves do not allow one to assess the *modelling robustness* introduced in §3, therefore whether the results found for one modelling framework generalize to other frameworks. One possibility is to generalize reaction–diffusion systems to include stochastic effects. Two possibilities to achieve this are either by means of stochastic partial differential equations maintaining continuous space and concentrations [40], or by means of the reaction–diffusion master equation which discretizes space and models chemical species by discrete molecule counts [41,42]. Several studies have found that models in either approach can give rise to patterns outside of the parameter Turing space found in deterministic models, and hence to an increased parametric robustness [28,43–47]. These results suggest that real biological networks might be more robust than assumed from deterministic models. It remains to be seen, however, to what extent these results generalize beyond single case studies.

Lattice gas cellular automata (LGCA) constitute an alternative modelling framework to the deterministic and stochastic reaction–diffusion processes described above. Originally developed to simulate fluid flow in the 1970s, LGCA models discretize space and time, and allow chemical concentrations to only access a finite number of discrete states [48] (figure 2). Here, discrete reaction maps define the system rather than continuous kinetic parameters. In [49,50], they have been used to study TPs for single reaction networks. Recently, the parametric robustness of networks has been investigated in [20] where a comprehensive analysis of all 2-node networks and a certain class of reaction maps has been conducted. One important finding is that the LGCA model identifies the same five 2-node topologies as found in the continuous case [11] (figure 2). This is a prime example of modelling robustness, where a fundamental result is shared between distinct modelling frameworks, and stresses the fundamental nature of the Turing mechanism. The authors in [20] analysed the robustness of networks in terms of fractions of reaction maps and found surprisingly large robustness values. While these cannot be directly compared to robustness values found in continuous systems in terms of fractions of parameter spaces, this may still suggest that Turing systems are more robust than previously thought.

**Figure 3.**
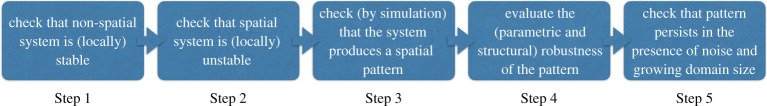
Checklist for rational design of Turing-patterning systems. Each system should pass these five tests. Unfortunately, the existence of a Turing stability is not sufficient to guarantee the existence of a pattern, which therefore needs to be tested separately (Step 3) [11]. Even if a pattern exists, it is important to check that the amplitude of the morphogen pattern is physiologically relevant. (Online version in colour.)

In practical terms, for the rational design of TPs—that is, using mathematical or computational triaging of suitable candidate models—we need to employ careful checks to safeguard that a model really satisfies all that is required to produce a TP. To be a good candidate for an *in vivo* TP-generating mechanism, a model should pass through the five steps shown in figure 3.

On top of that there will, of course, be other considerations [6], typically to do with the ability to synthesize system components, and ensuring that they operate orthogonally to the cellular machinery; but this seems increasingly possible to achieve with due care [17,51].

## 5. Turing patterns versus positional information

It is worthwhile to briefly revisit the widely perceived dichotomy between TP and PI mechanisms and their biological roles. There are good biological reasons why these two mechanisms cannot explain all developmental processes in isolation. Furthermore, as explained by Green & Sharpe [8], there is a need to consider both approaches working together: many phenomena are best explained if both processes are tightly coupled. An easy way to see this is to use the TP mechanism to give rise to the spontaneous symmetry-breaking by which a gradient will form, and this gradient can then be interpreted by a mechanism that embodies the hallmarks of the PI model.

Here, however, we are interested in the mathematical nature of the models involved in generating TPs or PI. Historically, PI has often not been explicit about mechanistic models, often resorting to purely verbal models [2]. There are also, compared to the TP case [11,22,23], few studies investigating design principles of PI mechanisms. One important study of stripe formation [52] found a set of network architectures capable of producing stripes. One fundamental question that has so far received only limited attention is whether the networks capable of producing TPs are fundamentally distinct from those capable of producing PI patterns; or if they are in fact related.

**Figure 4.**
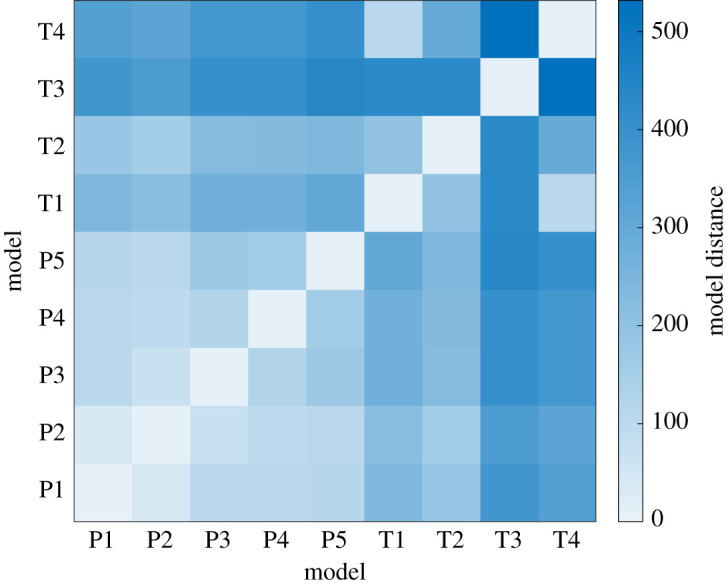
Distance matrix calculated from simplicial complex representations of positional information models (P1–P5) and TP models (T1–T4), where distances are taken from [54]. The figure shows that published models from the two mechanisms do not form distinct subgroups. T2, for example, is closely related to P1 and P2, indicating that there are perhaps less dramatic differences between the two mechanisms, or at least the mathematical models describing these mechanisms, than may have been previously believed. (Online version in colour.)

**Figure 5.**
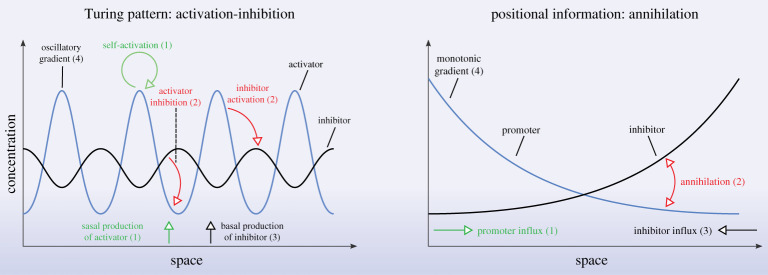
Comparison of the Turing-pattern activator-inhibitor model and the positional-information annihilation model. Model components that are identified as equivalent between the two models are indicated with numbers in parentheses. (Online version in colour.)

At the level of network structure—which by itself does not suffice to constrain the system dynamics [53]—there appears to be some overlap in the motifs found in different contexts [11,52]. A more thorough analysis of different detailed mechanistic models [54], however, suggests that there are indeed considerable similarities between models producing TPs and models embodying PI. Indeed, we can represent models as labelled simplicial complexes by including all model components, such as molecular species, diffusion, types of interactions, boundary conditions, and global aspects of the solutions, as vertices, with edges and higher-dimensional simplices representing interconnections between the model components, such as an edge between a molecular species and its diffusion. It is then possible to construct a distance function between pairs of labelled simplicial complexes, corresponding to the total number of simplices that occur in only one of the two simplicial complexes, that provides a measure of conceptual similarity between the models, so that a larger distance corresponds to a greater conceptual difference. Such analysis shows that some of the models previously reported to be TP mechanisms are in fact closely related to models proposed as generating PI patterns (and vice versa). Indeed, in figure 4, we compare the distances between five PI models and four TP models. The PI models are the linear gradient, synthesis-diffusion-degradation, opposing gradients, annihilation, and scaling by modulation models, referred to as P1–P5, respectively; and the TP models are the activator-inhibitor, substrate depletion, inhibition of an inhibition, and modulation models, referred to as T1–T4, respectively. In some cases, there are even deeper relationships between models, which can be revealed by identifying components between the models that are conceptually equivalent, and then looking for corresponding transformations of the associated simplicial complexes [54]. In particular, applying this methodology to the TP activator-inhibitor model and the positional-information annihilation model reveals that the two models are, at least from a mathematical perspective, equivalent and effectively identical. We illustrate this equivalence graphically in figure 5. The upshot of this analysis is that we may able to consider mechanistic models in development in a more nuanced way, as already pointed out for biological reasons in [8], and stress the similarities and shared principles.

## 6. Conclusion

Feynman’s famous statement ‘What I cannot create I do not understand’^1^ appears to have a corollary: what we do not understand we cannot build. Certainly as far as TPs are concerned. There are a host of caveats that need to be considered in designing a synthetic Turing-patterning mechanism that is viable *in vivo*.

Here, we have touched upon issues related to robustness. From our discussion, we hope, it is clear that much work remains to be done, both in understanding fundamental properties of TPs and their design principles, and as regards to putting notions of robustness on a solid mathematical footing. For a rational synthetic biology, one based on engineering design methods prior to implementation, these two facets are intricately linked.

The set of recent large-scale analyses into TP design principles [6,20,22,23,55] (and similar attempts at elucidating other design principles [52,56,57]) can help to distil design principles of cellular function, and guide the synthetic biology design process. However, as discussed above, great care needs to be taken to set up such computationally demanding analyses. Put bluntly, performing of the order of $10^{6}$ linear stability analyses will throw up a potentially large number of cases where numerical problems occur and we need to capture and treat such cases separately. Automating such mathematical analyses is demanding, but necessary in order to get the correct answers (as outlined above).

Being able to draw on this recent body of work it is possible to speculate (with increasing confidence) on what good candidates for TPs will look like (§4). Moreover, based on similar reasoning, but drawing on concepts from simplicial complexes and persistent homology [54,58], it is possible to show perhaps unexpected similarities—and, indeed, equivalences—between models of types of processes that had previously been considered separately.

What this shows, in summary, is that systems and synthetic biology can benefit enormously from moving away from the analysis of single models, or small sets of models to consider much larger sets of models. While challenging, we believe that such a perspective will be more helpful in studying robust design features of real and synthetic biological systems, than the reliance on detailed analysis of single models.

## Footnotes

1 Written on his blackboard at the time of his death.

## Data accessibility

This article has no additional data.
